# Cathelicidin and Calprotectin Are Disparately Altered in Murine Models of Inflammatory Arthritis and Airway Inflammation

**DOI:** 10.3389/fimmu.2020.01932

**Published:** 2020-08-20

**Authors:** Mahadevappa Hemshekhar, Hadeesha Piyadasa, Dina Mostafa, Leola N. Y. Chow, Andrew J. Halayko, Neeloffer Mookherjee

**Affiliations:** ^1^Department of Internal Medicine, Manitoba Centre for Proteomics and Systems Biology, University of Manitoba, Winnipeg, MB, Canada; ^2^Department of Immunology, University of Manitoba, Winnipeg, MB, Canada; ^3^Department of Physiology and Pathophysiology, University of Manitoba, Winnipeg, MB, Canada; ^4^Biology of Breathing Group, The Children's Hospital Research Institute of Manitoba, Winnipeg, MB, Canada

**Keywords:** inflammation, cathelicidin, calprotectin, host defence peptides, antimicrobial peptides, arthritis, asthma, airway

## Abstract

Cationic host defense peptides (CHDP) are immunomodulatory molecules that control infections and contribute to immune homeostasis. CHDP such as cathelicidin and calprotectin expression is altered in the arthritic synovium, and in the lungs of asthma and COPD patients. Recent studies suggest a link between airway inflammation and the immunopathology of arthritis. Therefore, in this study we compared the abundance of mouse cathelicidin (CRAMP), defensins, and calprotectin subunits (S100A8 and S100A9) in murine models of collagen-induced arthritis (CIA) and allergen house dust mite (HDM)-challenged airway inflammation. CRAMP, S100A8, and S100A9 abundance were significantly elevated in the joint tissues of CIA mice, whereas these were decreased in the lung tissues of HDM-challenged mice, compared to naïve. We further compared the effects of administration of two different synthetic immunomodulatory peptides, IG-19 and IDR-1002, on cathelicidin and calprotectin abundance in the two models. Administration of IG-19, which controls disease progression and inflammation in CIA mice, significantly decreased CRAMP, S100A8, and S100A9 levels to baseline in the joints of the CIA mice, which correlated with the decrease in cellular influx in the joints. However, administration of IDR-1002, which suppresses HDM-induced airway inflammation, did not prevent the decrease in the levels of cathelicidin and calprotectin in the lungs of HDM-challenged mice. Cathelicidin and calprotectin levels did not correlate with leukocyte accumulation in the lungs of the HDM-challenged mice. Results of this study suggest that endogenous cathelicidin and calprotectin abundance are disparately altered, and may be differentially regulated, within local tissues in airway inflammation compared to arthritis.

## Introduction

Cationic host defense peptides (CHDP), also known as antimicrobial peptides, are endogenous molecules that orchestrate host immune responses to infection and inflammation (1, 2). Immunity-related functions of CHDP include enhancing leukocyte migration and innate immune responses to resolve infections, regulation of endotoxin- and/or specific cytokine-induced inflammatory responses, promotion of the maturation and differentiation of leukocytes, induction of anti-inflammatory cytokines, contributing to tissue remodeling or wound healing, and overall maintenance of immune homeostasis (1–3). CHDP bridge innate and adaptive immune responses and play a role in promoting initiation, polarization, and amplification of adaptive immunity (1). These peptides are expressed by both immune and structural cells, and expression levels of some CHDP are enhanced in presence of infection or inflammatory challenge. Several studies have demonstrated altered levels of CHDP cathelicidin and calprotectin (a complex of subunits S100A8 and S100A9) in various chronic inflammatory diseases including rheumatoid arthritis (RA), chronic obstructive pulmonary disease (COPD), asthma, inflammatory bowel disease (IBD) and atherosclerosis (3–12). However, alteration of abundance of specific CHDP in chronic inflammatory diseases has not been completely defined.

Recent evidence suggests the involvement of airway inflammation to the onset and the immunopathology of RA (13, 14). Environmental factors such as smoking and air pollution that lead to the exacerbation of airway inflammation are also critical risk factors for the development of RA (13). Thus, although airway inflammation and inflammatory arthritis have distinct phenotype, these exhibit overlapping molecular mechanisms of immune dysregulation. A mechanism linking airway inflammation to RA is the production of antibodies directed against citrullinated peptide targets (anti-CCP). These antibodies are found in the lungs during airway inflammation and are defined as autoantibodies in RA (15, 16). Interestingly, CHDP such as LL-37 can get citrullinated in airway inflammation, which alters the functions of the peptide (17). Moreover, the abundance of circulating LL-37 is associated with anti-CCP in early RA (18). However, the role of CHDP in respiratory inflammatory disease progression and in the immunopathology of RA remains elusive. We have previously shown that inhaled air pollution, which is a critical environmental risk factor for both airway inflammation and the development of RA, alters the expression profile of CHDP in the human lungs (19). We have also shown that cytokines that are elevated during inflammation in both the lungs and joints, such as IL-17 and TNF, can significantly change the expression profile of CHDP in epithelial cells (20). Therefore, in this study we examined *in vivo* the expression of a panel of CHDP selected from these previous studies, in tissues isolated from murine models of airway inflammation and arthritis. The objective was to examine if specific endogenous CHDP are altered similarly within the local tissues in these two interconnected disease processes.

It is unknown whether change in the abundance of endogenous CHDP during inflammation is solely related to enhanced accumulation of inflammatory leukocytes within the local tissues. Synthetic peptides based on CHDP sequence have been previously shown to mitigate local inflammation in chronic inflammatory disease models (2). For example, the human cathelicidin LL-37-derived peptide IG-19, which represents the minimum region of LL-37 required for immunomodulatory functions (21), can suppress pro-inflammatory cytokine secretion (22, 23), and reduce leukocyte accumulation and control inflammatory mediators in the joints in a collagen-induced arthritis (CIA) mouse model (24). Similarly, a bovine cathelicidin-derived peptide IDR-1002 mitigates airway inflammation, controls leukocyte accumulation in the lungs, and improves airway hyper-responsiveness (AHR) in an allergen house dust mite (HDM)-challenged mouse model (25). Therefore, in this study we used the synthetic immunomodulatory peptides IG-19 and IDR-1002 as probes to examine whether the change in the abundance of endogenous CHDP is associated with enhanced inflammation within the local tissues. The hypothesis being that if CHDP abundance is altered solely due to enhanced inflammation such as that mediated by the influx of inflammatory leukocytes within the local tissues, then the administration of the specific anti-inflammatory synthetic peptides would result in the altered CHDP levels being reversed and/or restored to baseline, in both models.

In this study, we investigated the abundance of the mouse cathelicidin CRAMP, α - and β-defensins, and calprotectin subunits S100A8 and S100A9, in the two different murine models of chronic inflammation; a CIA model of inflammatory arthritis and allergen HDM-challenged model of airway inflammation (25–27). We showed that CRAMP and calprotectin subunits S100A8, and S100A9 are disparately altered in these mouse models, being significantly increased in the joint tissues of CIA mice, but decreased in the HDM-challenged lungs. Overall, the results in this study suggest that endogenous CHDP cathelicidin and calprotectin abundance are altered disparately, and may be differentially regulated within local tissues in airway inflammation compared to inflammatory arthritis.

## Methods

### Collagen-Induced Arthritis (CIA) Murine Model

The protocol used for the CIA murine model was based on our previous study (26) and approved by the University of Manitoba Animal Research Ethics Board (protocol no 16-009). ARRIVE guidelines were followed in designing and reporting animal data (28). Briefly, highly susceptible DBA/1 male mice (~6 weeks old) were obtained from Jackson laboratories, sorted into 4 mice per cage by animal care staff and allowed to acclimatize to the facility for 2-weeks at the central animal care facility at University of Manitoba. Mice were anesthetized using isoflurane (4%) and challenged with a tail injection (s.c) of 100 μg bovine collagen type II (CII) emulsified in complete Freund's adjuvant. A boost of CII emulsified (50 μg) in incomplete adjuvant was administered on day 21 after the initial CII challenge. On day 25 after the first CII challenge, mice were injected intra-peritoneally (i.p.) with LPS from *E. coli* 0111:B4 (20 μg per mouse). Administration of low dose of LPS synchronizes the disease incidence in the CIA model, and therefore allows for accurate comparative evaluation of outcomes between different groups (26, 29, 30). Collagen and LPS challenge were performed between 10:00 am and noon. All reagents for the CIA challenge were obtained from Chondrex Inc. (Redmond, WA, USA). Mice with saline injections were used as the control group. Mice were monitored for change in body weight every alternate day, and were visually monitored for grooming and activity levels every day. Joint thickness was monitored daily using a digital caliper from day 22 onwards to assess disease progression as described previously (24, 26). Disease severity was assessed in a blinded manner using a standardized clinical score based on joint thickness / swelling data, as previously described by us (24, 26). Briefly, clinical score assessment was as follows: Score 0 = normal joint; 1 = paw swelling only; 2 = one joint of one limb along with paw swelling; 3 = multiple joints on a limb involved; and 4 = all joints involved or limb fusion. Therefore, a total clinical score ranging from 0 to 16 was assigned to each mouse by combining the scores of each paw (24, 26). On day 29 after the first CII challenge, mice were anesthetized with isoflurane (4%) and euthanized by cardiac puncture, blood collected and used to obtain serum samples. Serum obtained was aliquoted and stored in −20°C until use. Mice joints were collected, cleaned to remove skin/tissues and homogenized to prepare protein lysates.

### HDM-Induced Allergic Asthma Mice Model

The protocol used for the HDM murine model was based on our previous study (25, 27) and approved by the University of Manitoba Animal Research Ethics Board (protocol no 16-040). ARRIVE guidelines were followed in designing and reporting animal data (28). Briefly, female BALB/c mice (6–7 weeks) were obtained from the Genetic Modeling of Disease Center at the University of Manitoba. Mice were sorted into 4 mice (*n* = 4) per cage by animal care staff and housed at the central animal care facility at University of Manitoba. Animals were acclimatized for a minimum of 1 week. Mice were sedated using isoflurane and challenged with intranasal (i.n) administrations of 35 μL of HDM extract (0.7 μg/mL of saline) or saline, 5 times a week for 5 weeks. HDM extracts used in this study were obtained from Greer laboratories (Wilmington, MA, USA), with low endotoxin levels between 600 and 800 EU/vial (which is 115 and 260 EU/mg of protein weight). HDM challenge was performed in the morning between 10:00 a.m. and noon. Mice were visually monitored for grooming and activity levels every day. Mice were sacrificed 24 h after the last HDM-challenge based on our previous study (25), and right lower lobe lung tissues collected for protein analyses.

### Administration of Cathelicidin-Derived Synthetic Peptides in Mice

Peptides IG-19 and IDR-1002 were obtained from CPC Scientific (CA, USA) (21, 23, 25, 31). IG-19 (IGKEFKRIVQRIKDFLRNL-NH_2_) is derived from the human cathelicidin LL-37 (amino acids 13-31) (21, 23). Innate Defense Regulator (IDR) peptide, IDR-1002 (VQRWLIVWRIRK-NH2), is derived from bovine cathelicidin Bac-2A (25, 31). These synthetic peptides were re-suspended in sterile saline and administered by s.c. injections (6 mg/kg body weight) in both models. The dose and mode of administration of these peptides were based on our previous studies in the CIA model and HDM-challenge model (24–27). In the CIA model, the peptides were administered starting from day 20 [1 day before boost, after the initial arthritis induction phase (32)] and subsequently every 48 h until the end of the study. As the anti-inflammatory effect of the peptide IG-19 was previously demonstrated in the CIA model without LPS boost (24), we also tested the peptide IDR-1002 in the CIA model without LPS boost. In the HDM-challenge model, the peptides were administered 3 days per week starting from day 1, for 2 weeks. Peptide treatments were performed between 10:00 a.m. and noon.

### Tissue Sample Preparation

After the mice were euthanized, joint tissues were collected from mice in the CIA model, and lung tissues were obtained from mice in the HDM-challenge model, as described above. The tissues were flash frozen in liquid nitrogen and stored in −80°C until further use. The flash frozen tissues were homogenized on ice using a tissue homogenizer (Omni International, USA), in protein extraction buffer T-PER (Thermo Scientific, USA) containing protease inhibitor cocktail (Cell Signaling Technology, Denver, USA). The homogenates were centrifuged at 10,000 × g, at 4°C for 10 min. The supernatants were collected, aliquoted and stored in −20°C until use. Total protein amount was estimated in the supernatants using micro-Bicinchoninic acid (BCA) assay (Thermo Scientific, USA) according to the manufacturer's instructions.

### Immunoblotting

The joint and lung tissue lysates (20 μg per sample) were resolved on NuPage 4–12% Bis-Tris protein gels (Invitrogen) and transferred onto nitrocellulose membranes. The membranes were blocked overnight with 5% milk powder (w/v) and probed with antibodies for murine CRAMP (rabbit polyclonal, Abcam, USA, catalog number ab93357), S100A8 (rat monoclonal [clone ABM4A69], Abcam, catalog number ab220174), S100A9 (rat monoclonal [clone 2B10], Abcam, catalog number ab105472), α-defensin 1 (goat polyclonal, Abcam, catalog number ab122884), β-defenisn 2 (rabbit polyclonal, MyBioSource, USA, catalog number MBS2005685) and β-defensin 14 (rabbit polyclonal, MyBioSource, catalog number MBS1490249). Antibody to β-actin (Cell Signaling Technologies) was used to normalize for protein loading. Affinity-purified horseradish peroxidase (HRP)-linked secondary antibodies (Cell Signaling, USA) along with Amersham ECL Prime (GE Healthcare) was used for detection. The blots were imaged using AmershamTM Imager 680 blot and gel imager. Densitometry assessment of band intensity was determined using AmershamTM Imager 680 analysis software version 2.0. The relative band intensity was assessed after normalization with the band intensity for β-actin.

### Evaluation of Anti-collagen Type II (CII) Antibodies in Serum

Circulating levels of mouse anti-collagen antibodies (auto-antibodies) and bovine anti-collagen antibodies was assessed by ELISA in serum collected from mice in the CIA model. A mouse anti-mouse type II collagen IgG antibody assay kit and mouse anti-bovine type II collagen IgG antibody assay kit was used, according to the manufacture's protocol (Chondrex Inc. Redmond, WA, USA). The antibody concentrations in the samples were calculated by comparison with the optical density (OD) values of standard anti-CII antibody (units/ml) provided in each of the assay kits as previously described (24, 26).

### Cell Differential Assessment in Bronchoalveolar Lavage Fluid (BALF)

Mice in the HDM-challenged model were anesthetized with sodium pentobarbital (90 mg/kg, i.p), tracheotomised, and lungs were washed with 1 mL of cold saline twice for a total of 2 mL. BALF obtained was centrifuged at 150 × g for 10 min at room temperature and cell differentials were assessed using a modified Wright-Giemsa staining (Hema 3® Stat Pack, Fisher Scientific, Hampton, NH, USA) using a Carl Zeiss Axio Lab A1 (Carl Zeiss, Oberkochen, Germany) microscope, as previously described (25, 27). Cell differentials were counted blinded by two different personnel in 5 image frames at 20X magnification per slide, as previously described by us (25).

### Lung Function Measurements

Mice from the HDM-challenged model were anesthetized with sodium pentobarbital (90 mg/kg, i.p.) and tracheostomized. Prior to lung function testing, mice received additional 45 mg/kg pentobarbital to maintain a stable anesthetized state. Lung function measurements was performed using a *flexi*Vent™ small animal ventilator (SCIREQ Inc, Montreal, QC, Canada) as previously described (33, 34). Briefly, high frequency forced oscillation with positive end-expiratory pressure of 3 cmH_2_O was used to assess Newtonian resistance (Rn) to monitor central airway constriction, tissue damping (G) as an index of alveolar tissue restriction, and tissue elastance (H) to determine alveolar tissue stiffness. A muscle paralytic agent was not used in this method. Data was collected using flexiWare Software and transferred to Microsoft Excel and GraphPad Prism software for further analysis. Changes in Rn, G and H were monitored in response to nebulized saline (baseline measures), followed by increasing concentrations of nebulized methacholine (3–50 mg/mL), using Quick Prime-3 and Snapshot perturbations (25).

### Histological Assessment of Joints for Cellular Infiltration

The ankle joints of mice were collected on the day of sacrifice and fixed in 10% buffered formalin for 48 h. The joints were decalcified using 10% EDTA for 14 days followed by dehydration in increasing concentrations of ethanol. The tissues were embedded in paraffin and serial sagittal sections (5 μm) were obtained. The sections were stained with hematoxylin and eosin (H&E) to assess influx of leukocytes in the joints. Sections were imaged and processed with a Zeiss imager M2 (Germany) using the Zen 2011 software. The stained sections were scored as previously described (24, 26) in a blinded manner by three independent personnel. Briefly, a histology score to assess the leukocyte infiltration and integrity of the joints was as follows: A score of 0 = normal synovium, 1 = synovial membrane hypertrophy and cell infiltration, 2 = pannus formation and cell infiltration, 3 = joint degeneration and robust cell infiltration, and 4 = loss of joint integrity along with robust cellular infiltration (24, 26).

### Statistical Analysis

GraphPad Prism 7.05 software was used for data analyses. Statistical significance was determined using Kruskal–Wallis One-way analysis of variance (ANOVA) followed by Dunn's *post-hoc* test when comparing three or more groups. Mann–Whitney *U*-test was used to determine the *p*-values between any two groups as presented in Table 1. Pearson's correlation analysis was performed to examine the correlation between CHDP abundance with joint histology score in the CIA model, or total cell count in the BALF in the HDM model. A *p*-value of < 0.05 was considered to be statistically significant.

**Table 1:** Change in the relative abundance of CRAMP and calprotectin in murine models of CIA and HDM-challenged airway inflammation.

	**Relative fold change in joint tissues compared to saline (densitometry)**				**Relative fold change in lung tissues compared to saline (densitometry)**			
	**CIA**	**CIA+IG-19**	**% change**	***p*-value**	**HDM**	**HDM+IDR1002**	**% change**	***p*-value**
CRAMP	75 ± 27	8 ± 1.3	89 ± 1.28%	0.028	0.36 ± 0.1	0.49 ± 0.16	22.6 ± 7.1%	0.48 ns
S100A8	688 ± 252	95 ± 34	86 ± 1.82%	0.028	0.29 ± 0.08	0.48 ± 0.12	31.4 ± 16%	0.34 ns
S100A9	593 ± 350	58 ± 25	90.7 ± 6.2%	0.028	0.30 ± 0.1	0.31 ± 0.11	1.3 ± 9%	0.99 ns

*ns, non-significant*.

## Results

### Endogenous CRAMP and Calprotectin Abundance Are Altered in CIA and HDM-Challenged Mice

**Figure 1:**
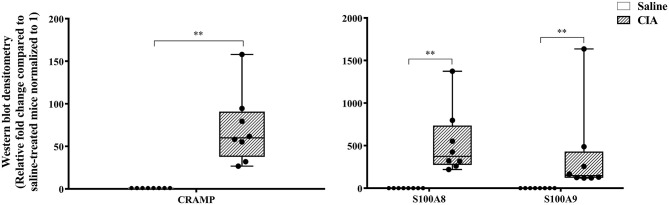
CRAMP and Calprotectin levels are elevated in murine model of CIA. Flash frozen tissues obtained from joints of the CIA model (*n* = 8) and saline control (*n* = 8) were homogenized and protein concentration was determined using miroBCA. The samples (20 μg protein each) were resolved on NuPage 4–12% Bis-Tris protein gels and transferred onto nitrocellulose membranes. The membranes were probed with antibodies for mouse CRAMP, S100A8, S100A9 and β-actin (loading control). Densitometry for band intensity was determined using AmershamTM Imager 680 analysis software version 2.0. The relative band intensity was determined by normalizing to the β-actin band intensity for each sample. The represented values are the relative fold change compared to saline-treated mice normalized to 1. GraphPad Prism 7.05 software was used for statistical analyses. Mann–Whitney *U*-test was used to determine the *p*-values. A *p*-value of < 0.05 was considered to be statistically significant (**p ≤ 0.005).

**Figure 2:**
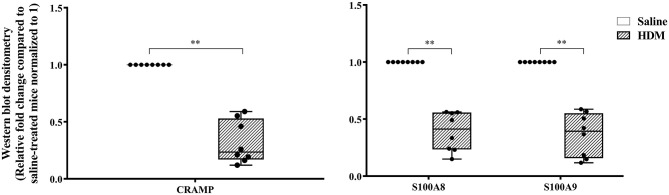
CRAMP and Calprotectin levels are decreased in murine model of HDM-challenge-induced airway inflammation. Flash frozen tissues obtained from lungs of HDM-challenge model (*n* = 8), and saline control (*n* = 8) were homogenized and protein concentration was determined using miroBCA. The samples (20 μg protein each) were resolved on NuPage 4–12% Bis-Tris protein gels and transferred onto nitrocellulose membranes. The membranes were probed with antibodies for mouse CRAMP, S100A8, S100A9 and β-actin (loading control). Densitometry for band intensity was determined using AmershamTM Imager 680 analysis software version 2.0. The relative band intensity was determined by normalizing to the β-actin band intensity for each sample. The represented values are the relative fold change compared to saline-treated mice normalized to 1. GraphPad Prism 7.05 software was used for statistical analyses. Mann-Whitney U test was used to determine the *p*-values. A *p*-value of < 0.05 was considered to be statistically significant (**p ≤ 0.005).

The abundance of CRAMP, S100A8, S100A9, α-defensin 1, β-defensin 2 and β-defensin 14 were examined by western blots in the joint tissue lysates obtained from CIA and saline-treated control mice, and in the lung tissue lysates obtained from HDM-challenged and allergen-naïve mice. Defensins were not detected in measureable abundance in the joint or lung tissue lysates. CRAMP, S100A8, and S100A9 were not detected in the joint tissue lysates of saline-treated control mice, whereas the abundance of these CHDP was robust in joint tissue lysates of CIA mice (Supplementary Figure 1). Densitometry analyses demonstrated that the levels of CRAMP, S100A8, and S100A9 peptides were significantly (*p* ≤ 0.002) increased between 70 and 500-fold in the joint tissues of CIA mice, compared to saline-treated mice (Figure 1). In contrast, the levels of CRAMP, S100A8, and S100A9 peptides were significantly (*p* ≤ 0.001) decreased by 65 ± 6.5%, 60 ± 6%, 62 ± 6.7%, respectively in the lung tissues of HDM-challenged mice, compared to allergen-naïve mice (Figure 2).

### Administration of LL-37-Derived Synthetic Peptide IG-19 Alters the Abundance of CRAMP and Calprotectin in the Joints of CIA Mice

**Figure 3:**
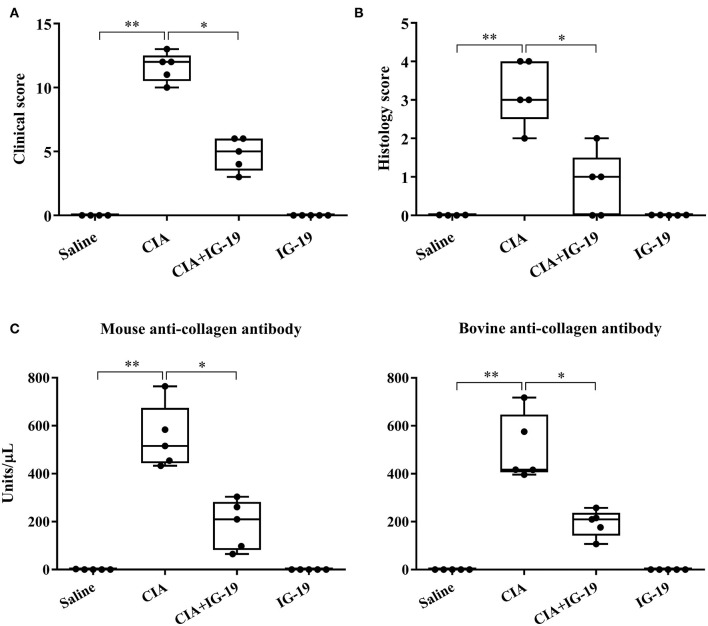
Administration of IG-19 reduces clinical scores and anti-collagen II antibody levels in CIA mice. DBA/1 male mice (8 weeks) were challenged (s.c.) with bovine CII (*n* = 4), followed by a booster dose of CII (s.c) on day 21 and a LPS boost (i.p.) on day 25 after the initial CII challenge. Peptide IG-19 was administered (s.c) every 48 h from day 20 after the first CII challenge (one day before boost). Mice were monitored for disease severity and assigned clinical scores from day of CII boost (day 21 after the first CII challenge), every alternate day. Mice were euthanized by cardiac puncture under anesthesia on day 29 after the first CII challenge and blood collected for serum. **(A)** Clinical scores, **(B)** histology scores, and **(C)** serum concentration of anti-mouse collagen II autoantibodies and anti-bovine collagen II antibodies (to the immunizing antigen), assessed on the day of sacrifice. GraphPad Prism 7.05 software was used for statistical analyses. Kruskal–Wallis One-way ANOVA followed by Dunn's multiple comparison test was used to determine the significance (*p ≤ 0.05, **p ≤ 0.01).

**Figure 4:**
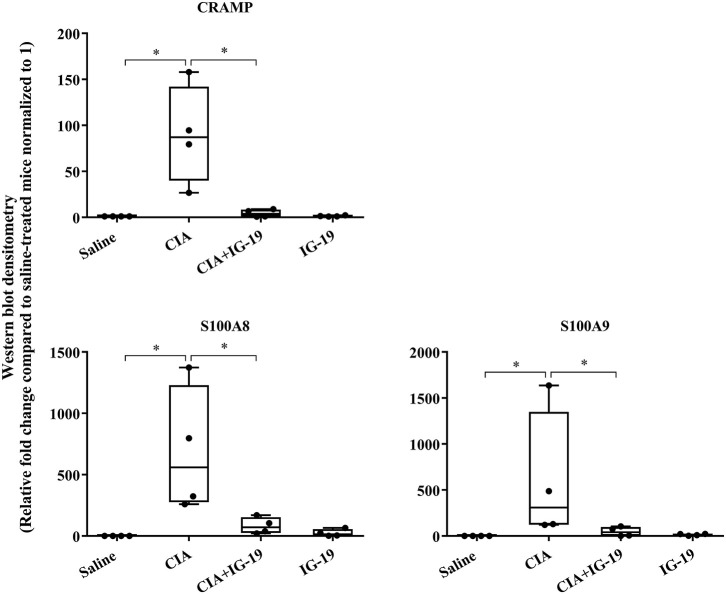
Administration of peptide IG-19 alleviates elevated levels of CRAMP and Calprotectin in joint tissues of CIA mice. Flash frozen tissues obtained from joints of saline treated (*n* = 4), CIA (*n* = 4), CIA mice administered with either peptide IG-19 (*n* = 5) or peptide alone (*n* = 5 each), were homogenized and protein concentration was determined using miroBCA. The samples (20 μg protein each) were resolved on NuPage 4–12% Bis-Tris protein gels and transferred onto nitrocellulose membranes. The membranes were probed with antibodies for mouse CRAMP, S100A8, S100A9 and β-actin (loading control). Densitometry for band intensity was determined using AmershamTM Imager 680 analysis software version 2.0. The relative band intensity was determined by normalizing to the β-actin band intensity for each sample. The represented values are the relative fold change compared to saline-treated mice normalized to 1. GraphPad Prism 7.05 software was used for statistical analyses. Kruskal–Wallis One-way analysis of variance (ANOVA) followed by Dunn's *post-hoc* test was used to determine the significance. A *p*-value of < 0.05 was considered to be statistically significant (*p < 0.05).

We have previously shown that administration (s.c.) of the human cathelicidin LL-37-derived peptide IG-19, from day 20 after initial CII challenge, prevents arthritic symptoms and suppresses anti-collagen antibodies, in CIA mice using a protocol without LPS boost (24). The CIA mouse model protocol as detailed in this study includes a low dose LPS boost which has been shown to synchronize the disease onset and progression in the CIA model (26). Low dose of LPS in the CIA model synchronizes the arthritic clinical symptoms without compromising the severity and other characteristic features of the disease, which allows for an accurate comparative evaluation of outcomes across different groups (26, 29, 30). In this study, we tested the effects of s.c. administration of IG-19 in the CIA model synchronized with LPS boost as detailed in the methods of this study, as well as in the HDM-challenged mouse model of airway inflammation. Administration of IG-19 significantly decreased the clinical and histology scores by >50%, and suppressed both anti-bovine collagen antibody (antibodies to immunizing antigen) and anti-mouse autoantibodies by > 60% in the CIA mice (Figure 3; Supplementary Figure 2). These results were consistent with our previous study using the non-synchronized (without LPS) CIA mouse model (24). Taken together, these results indicated that s.c. administration of IG-19 from 1 day before CII boost prevents the development of the disease, suppresses anti-collagen antibodies, and suppresses accumulation of leukocytes in the joints, in CIA mice (24, 26). In contrast, administration of IG-19 did not improve HDM-induced AHR, and did not reduce leukocyte accumulation in the lungs of HDM-challenged mice (Supplementary Figure 3). Therefore, we further evaluated the effects of administration of IG-19 on the abundance of CRAMP and calprotectin in joint tissues in the CIA model in this study. Administration (s.c.) of IG-19 significantly suppressed elevated levels of CRAMP, S100A8, and S100A9 by 89 ± 1.28%, 86 ± 1.82%, and 90 ± 6.2%, respectively in CIA mice, effectively restoring the elevated abundance of these CHDP to baseline levels (Figure 4, Supplementary Figure 4, Table 1).

### Administration Synthetic Peptide IDR-1002 Does Not Alters the Abundance of Endogenous CRAMP and Calprotectin

**Figure 5:**
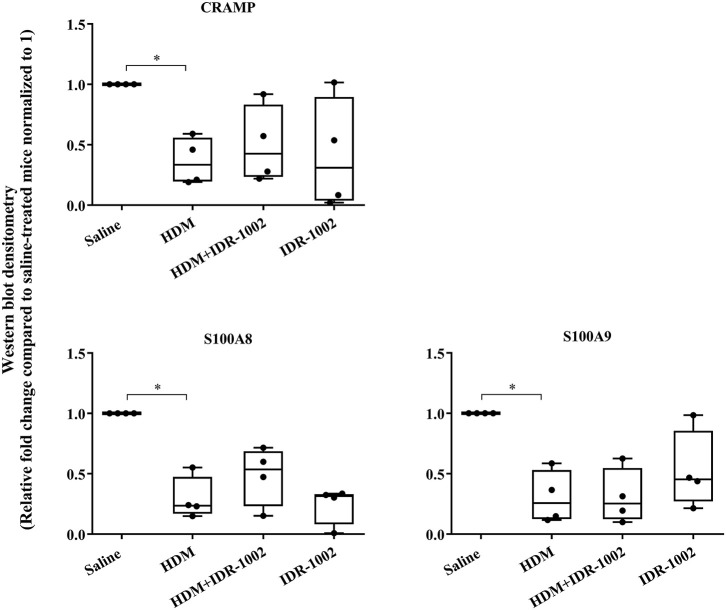
Administration of peptide IDR-1002 does not alter CRAMP and Calprotectin in lung tissues of HDM-challenged mice. Flash frozen tissues obtained from lungs of saline treated mice, HDM-challenged mice, HDM-challenged mice administered with peptide IDR-1002 or peptide alone (*n* = 4 mice per group), were homogenized and protein concentration was determined using miroBCA. The samples (20 μg protein each) were resolved on NuPage 4–12% Bis-Tris protein gels and transferred onto nitrocellulose membranes. The membranes were probed with antibodies for mouse CRAMP, S100A8, S100A9 and β-actin (loading control). Densitometry for band intensity was determined using AmershamTM Imager 680 analysis software version 2.0. The relative band intensity was determined by normalizing to the β-actin band intensity for each sample. The represented values are the relative fold change compared to saline-treated mice normalized to 1. GraphPad Prism 7.05 software was used for statistical analyses. Kruskal–Wallis One-way analysis of variance (ANOVA) followed by Dunn's *post-hoc* test was used to determine the significance. A *p*-value of < 0.05 was considered to be statistically significant (*p < 0.05).

We have previously shown that administration (s.c) of the bovine cathelicidin-derived synthetic peptide IDR-1002 alleviates airway inflammation and improves AHR in HDM-challenged mice (25). In contrast, administration of IDR-1002 did not prevent disease progression, and did not suppress anti-collagen antibodies in the CIA mice (Supplementary Figure 5). Therefore, we examined effect of administration (s.c.) of IDR-1002 on CRAMP and calprotectin abundance in lung tissues obtained from HDM-challenged mice. Administration of IDR-1002 peptide did not prevent the decrease of CRAMP, S100A8, or S100A9 in the lung tissues of HDM-challenged mice (Figure 5, Supplementary Figure 4, Table 1).

### Correlation of CRAMP and Calprotectin Abundance With Leukocyte Accumulation

**Figure 6:**
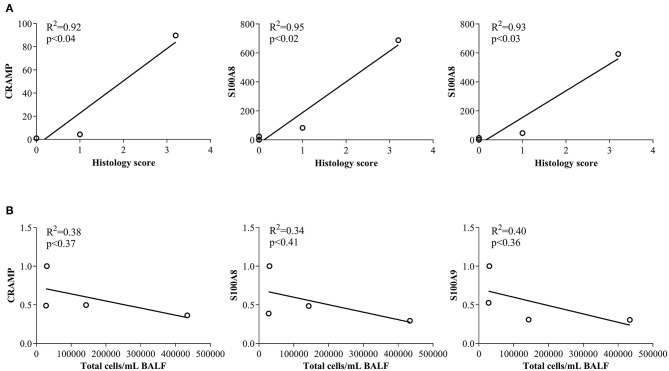
Correlation analyses of abundance of CRAMP and Calprotectin with cellular accumulation in local tissues. **(A)** Flash frozen tissues obtained from joints of saline treated (*n* = 4), CIA (*n* = 4), CIA mice administered with either peptide IG-19 (*n* = 5) or peptide alone (*n* = 5 each), were homogenized and used for western blots to examine the abundance of CRAMP, S100A8 and S100A9, using β-actin (loading control). Pearson's correlation analysis was performed to determine the correlation between the abundance of each peptide in the joints with histology score. **(B)** Flash frozen tissues obtained from lungs of saline treated mice, HDM-challenged mice, HDM-challenged mice administered with peptide IDR-1002 or peptide alone (*n* = 4 mice per group), were homogenized and used in western blots to examine the abundance of CRAMP, S100A8, S100A9, using β-actin (loading control). Pearson's correlation analysis was performed to determine the correlation between the abundance of each peptide in the lung tissues with total leukocyte count in the BALF. A *p*-value of < 0.05 was considered to be statistically significant.

In this study, we have shown that abundance of endogenous CRAMP and calprotectin subunits are altered in the joints of CIA mice and in the lungs of HDM-challenged mice (Figures 1, 2, respectively). Administration of immunomodulatory peptides IG-19 and IDR-1002, which controls inflammation in CIA mice and HDM-challenged mice, respectively (24, 25), decreased the enhanced levels of CHDP back to baseline in the CIA mice (Figure 4), but did not alter CHDP levels in the HDM-challenged mice (Figure 5). To determine whether change in the abundance of CRAMP and calprotectin is associated with inflammatory status within the local tissues, we performed correlation analyses between the abundance of CRAMP, S100A8, and S100A9 with histology score in the CIA model, and with total leukocyte count in BALF in the HDM model. Abundance of CRAMP, S100A8, and S100A9 in the joint tissues showed a significant linear correlation with histology scores in the CIA model (Figure 6A). In contrast, there was no significant correlation between the levels of CRAMP and calprotectin with total leukocytes in BALF in the HDM-challenged murine model (Figure 6B).

## Discussion

In this study, we demonstrated that the levels of mouse cathelicidin (CRAMP) and calprotectin (S100A8 and S100A9) were significantly increased in the joint tissues of CIA mice, a model that is used for preclinical studies of inflammatory arthritis (24, 26, 35). In contrast, CRAMP, S100A8, and S100A9 were significantly decreased in the lung tissues of allergen HDM-challenged mice, a model of airway inflammation that is used for preclinical studies of asthma (25, 27, 36). We further showed that administration of an immunomodulatory LL-37-derived synthetic peptide IG-19, which prevents disease progression and controls inflammation in CIA mice (24), significantly suppressed the elevated levels of CRAMP and calprotectin, restoring these to baseline levels in the joints of the CIA mice. However, administration of a bovine cathelicidin-derived synthetic peptide IDR-1002, which is known to alleviate HDM-induced airway inflammation and AHR (17), did not prevent the decrease of the levels of CRAMP or calprotectin from baseline in the lungs of HDM-challenged mice. Overall, the results in this study indicate that endogenous levels of CHDP cathelicidin and calprotectin are altered in local tissues in chronic inflammatory disease. However, whether these levels are enhanced or reduced depends on the disease type. Our results also suggest that changes in the abundance of endogenous cathelicidin and calprotectin may be directly related to the local inflammatory status in inflammatory arthritis, but not in airway inflammation.

Functions of cathelicidins and calprotectin in immunity and inflammation are well-documented (1–3, 37–41). For example, cathelicidins human LL-37 and mouse CRAMP are multifunctional immunomodulatory peptides that can act both as effector and regulator of inflammation (1, 3, 4, 42–44). These peptides mediate pro-inflammatory responses such as chemotaxis of immune cells, production of chemokines, and polarization and maturation of dendritic cells and T-lymphocytes (1, 3, 42). Cathelicidins can also suppress the inflammatory process by intervening in endotoxin or pro-inflammatory cytokine-induced signaling cascade, and by mediating anti-inflammatory responses such as activation of the dual phosphatase MKP-1 and production of anti-inflammatory cytokines IL-10 and IL-1RA (23, 43–47). Similarly, calprotectin is a calcium-binding protein, a heterodimer of subunits S100A8 and S100A9, that exhibits chemokine- and cytokine-like activity, initiate pro-inflammatory responses such as chemotaxis (37, 39, 41, 48), and also exhibit anti-inflammatory functions by acting as oxidant scavengers (48, 49). The wide repertoire of pro- and anti-inflammatory functions of cathelicidins and calprotectin has propelled interest in examining their role in chronic inflammatory diseases.

Several studies have noted that circulatory levels of LL-37 and calprotectin are altered in variety of chronic inflammatory diseases such as RA, COPD, asthma, cystic fibrosis, IBD, systemic lupus erythematosus and psoriasis (4, 37, 39, 41, 50, 51). We show in this study that cathelicidin and calprotectin are significantly enhanced in the joints in the CIA mice. Elevated levels of both LL-37 and calprotectin have been demonstrated in the serum and synovium of RA patients (6, 11, 52–54). Previous studies in pristane-induced models of arthritis have shown increase of CRAMP expressing cells in mouse (55), and enhanced levels of rat cathelicidin rCRAMP in the joints, blood and secondary lymphoid organs (11). Expressions of cathelicicidin and calprotectin subunit encoding genes were also shown to be elevated in CIA mouse model without low dose LPS boost (56). Taken together, these studies indicate that the enhanced abundance of calprotectin and cathelicidin in the joints reported here is not due to the low dose LPS injection in the CIA model, rather related to the inflammatory phenotype in the joints. Consistent with this, we showed that administration of an immunomodulatory synthetic peptide that reduces clinical scores and suppresses inflammation in the CIA mice, also decreased the elevated levels of cathelicidin and calprotectin restoring these to baseline levels in the joints of CIA mice. Moreover, there was a significant correlation between the abundance of cathelicidin and calprotectin with that of histology scores in the CIA mouse model. Therefore, the increase in the endogenous cathelicidin and calprotectin abundance in the joints is likely directly associated with the enhanced inflammatory phenotype in the joints in arthritis. However, how the enhanced CHDP levels relate to arthritis disease activity remains unclear. A study by Vogl et al. used optical imaging to demonstrate a significant correlation between increase in calprotectin subunit S100A9 and disease activity in the joints of CIA mice (57). Various studies have suggested a critical role of S100A8 and S100A9 in synovial activation and cartilage degradation during inflammatory arthritis (58–61). These evidences suggest that calprotectin may be involved in the activation and tissue degradation in arthritis. However, despite increase in cathelicidin being correlated with pathological changes in arthritis (11, 52, 53), cathelicidin-deficiency does not seem to affect the disease process in inflammatory arthritis (55). Thus, there is a lack of conclusive evidence for pathogenic role of cathelicidins in inflammatory arthritis. Nevertheless, results in our study corroborate previous findings that abundance of cathelicidin and calprotectin (S100A8 and S100A9) are significantly increased in the joint tissues, and that this is associated with increase in cellular influx indicative of increased inflammatory mediators in local tissues during the disease process in inflammatory arthritis. Contrary to this paradigm, we demonstrate in this study that endogenous levels of cathelicidin and calprotectin are decreased in the lung tissues of allergen HDM-challenged mice, which is a model of airway inflammation.

The HDM-challenged model results in increase in airway inflammation and AHR, and enhanced levels of various inflammatory proteins in the lungs (25, 27). Despite increase in airway inflammation in response to HDM challenge (27), here we show that CRAMP, S100A8, and S100A9 are significantly decreased in the lungs. We also showed that administration of the peptide IDR-1002, which has anti-inflammatory effects in the HDM-challenged mice, did not alter the abundance of the endogenous CHDP. Furthermore, there was no correlation between the levels of either cathelicidin or calprotectin with leukocyte accumulation in the BALF in the HDM-challenged mouse model. These results suggest there is no direct correlation with increase in inflammation and the change in endogenous CHDP cathelicidin and calprotectin levels in the lungs of HDM-challenged mice. Note that changes in cathelicidin and calprotectin levels reported from studies in diseases characterized by airway inflammation show considerable variability. A study showed an increase in LL-37 in BALF following segmental allergen challenge in adult allergic patients (62). In animal models of allergen challenge, mouse cathelicidin CRAMP was shown to be increased in the lungs in ovalbumin-challenged mice (63). In contrast, CRAMP was notably absent in the BALF of ovalbumin-sensitized mice infected with *Pseudomas aeruginosa*, despite significant increase in inflammatory cytokines (64). Such variability in cathelicidin levels is also observed in other diseases characterized with airway inflammation such as COPD. Plasma level of cathelicidin LL-37 is reduced in severe COPD patients (65, 66), but shown to be increased in sputum and blood in stable and in acute exacerbations of COPD patients (5, 65). A cross-sectional study by Golec et al. showed that concentration of LL-37 varied in BALF depending on the stage of the disease in COPD, significantly higher in early stages and lower in advanced disease stage, compared to healthy individuals (67). Similarly, studies examining calprotectin in airway inflammation show considerable variability. S100A8 gene expression is increased in blood samples obtained from children with asthma (68), and S100A9 is increased in the sputum of patients with asthma and COPD (69–71). These studies have suggested that the role of calprotectin is to amplify inflammation in respiratory disease. In contrast, other studies have suggested a protective role of calprotectin in airway inflammation, using exogenous administration of S100A8 and S100A9 in animal models of allergen-induced airway inflammation (72, 73). Therefore, how endogenous cathelicidin and calprotectin levels are altered, and their role in diseases characterized by airway inflammation remains ambiguous. The variability reported across studies may be due to the heterogeneity of processes related to immune dysregulation in airway inflammatory diseases. Results reported in this study demonstrating that endogenous levels of cathelicidin and calprotectin are differently altered in airway inflammation compared to inflammatory arthritis, may also be due to the heterogeneity in immune dysregulation within local tissues in these different diseases. Our results suggest that CHDP may be disparately regulated in airway inflammation compared to inflammatory arthritis. Although the immunopathology of airway inflammation (such as in asthma) and arthritis have been shown to be interconnected (13, 14), our results indicate that cathelicidin and calprotectin expression and related functions may not be one of the overlapping molecular mechanisms of immune dysregulation in these two disease processes. The mechanisms related to the differential regulation of CHDP in airway inflammation compared to arthritis warrants further investigation.

In summary, in this study we demonstrate that abundance of mouse cathelicidin (CRAMP) and calprotectin (S100A8 and S100A9) are inversely regulated in CIA and allergen HDM-challenged mice, being elevated in the joints but decreased in the lungs, respectively. We show that a synthetic immunomodulatory peptide that controls inflammation in CIA mice can also restore the elevated levels of cathelicidin and calprotectin to baseline in the joints. We also demonstrate that changes in these CHDP show a linear correlation with leukocyte accumulation in the joints in the CIA murine model. These results indicate that the enhancement of cathelicidin and calprotectin levels may be directly related to the inflammatory phenotype in the joints in arthritis. In contrast, we show that administration of an immunomodulatory peptide that controls airway inflammation cannot prevent the decrease in cathelicidin and calprotectin level in the lungs, and that the changes in these endogenous CHDP do not correlate with leukocyte influx in the lungs, in the HDM-challenged model. The results of this study indicate that the expression and/or regulation of CHDP within the local tissues in airway inflammation (e.g. in asthma) and arthritis are not similar, although the immunopathology of these diseases are known to be interconnected. Thus, the CHDP-mediated functions may not be among the common mechanisms of immune dysregulation in these two interconnected disease processes. Overall, the results of this study suggest that cathelicidin and calprotectin are disparately regulated in the local tissues in airway inflammation compared to inflammatory arthritis.

## Data Availability Statement

All datasets generated for this study are included in the article/Supplementary Material.

## Ethics Statement

The animal study was reviewed and approved by The University of Manitoba Animal Research Ethics Board.

## Author Contributions

MH, HP, and NM conceived the study and designed the experiments. MH, HP, DM, and LC performed the experiments and analyzed the data. NM directly supervised the study and obtained funding for reagents, materials, and analysis tools. MH wrote the manuscript. NM and AH extensively edited the manuscript. All authors reviewed the manuscript for submission.

## Conflict of Interest

NM is listed as an inventor on patents related to immunomodulatory aspects of host defense peptides and IDR peptides. The authors declare that the research was conducted in the absence of any commercial or financial relationships that could be construed as a potential conflict of interest.
