# Supplementary material for: Cathelicidin and Calprotectin Are Disparately Altered in Murine Models of Inflammatory Arthritis and Airway Inflammation
Source: Front Immunol. 2020 Aug 20;11:1932. doi: 10.3389/fimmu.2020.01932 (PMC7468387; doi:10.3389/fimmu.2020.01932)
Supplement: Supplementary file 1 [file Data_Sheet_1.PDF]

SUPPLEMENTARY INFORMATION

Supplementary Figure 1:

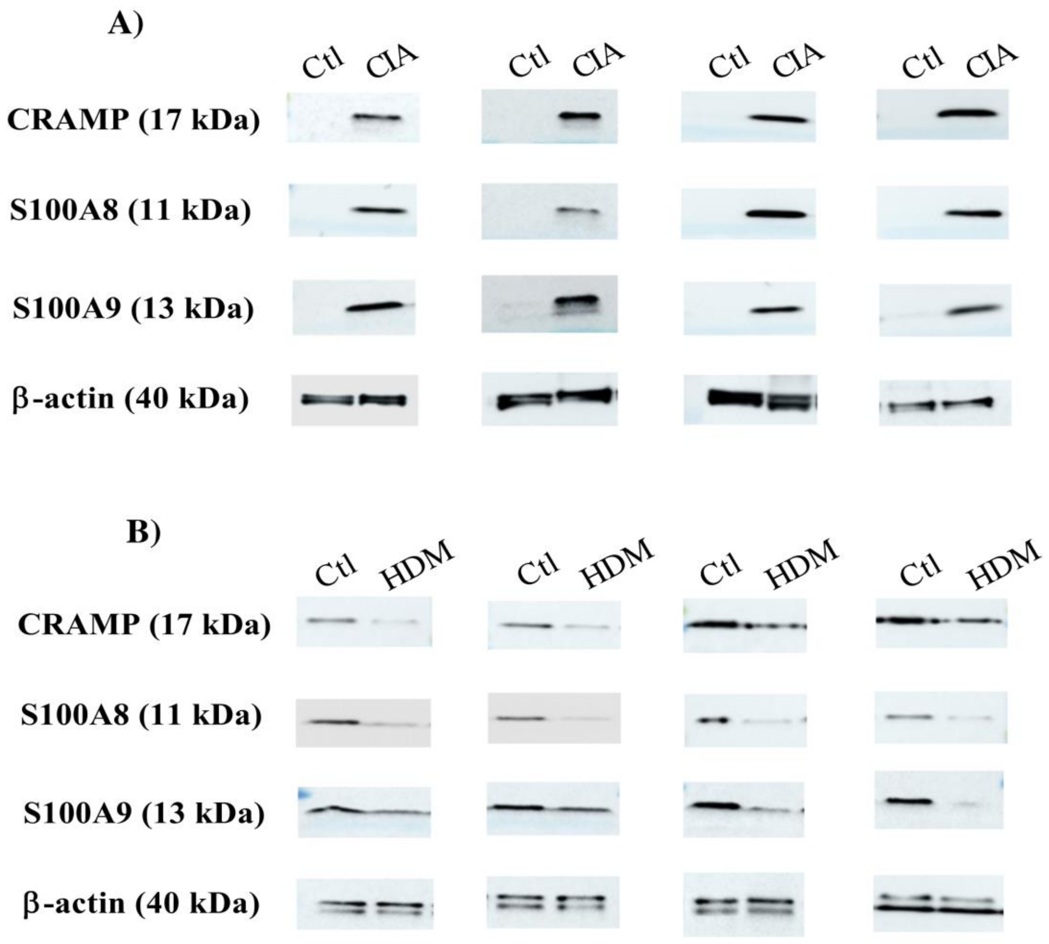

Figure S1: Western blots for abundance of CRAMP, S100A8 and S100A9, in murine models of CIA and HDM-challenge. Flash frozen tissues were obtained from **(A)** joints of mice from the CIA model (n=4 in each group), **(B)** lungs of HDM-challenge model (n=4 in each group), where control is saline-treated mice (Ctl). Tissues were homogenized and protein concentration determined using microBCA. Each sample (20 μg protein each) were resolved on NuPage 4-12% Bis-Tris protein gels and transferred onto nitrocellulose membranes. The membranes were probed with specific antibodies for mouse CRAMP, S100A8 and S100A9, and antibody for β-actin (loading control). The figure includes all 4 independent samples from each group for each model.

Supplementary Figure 2:

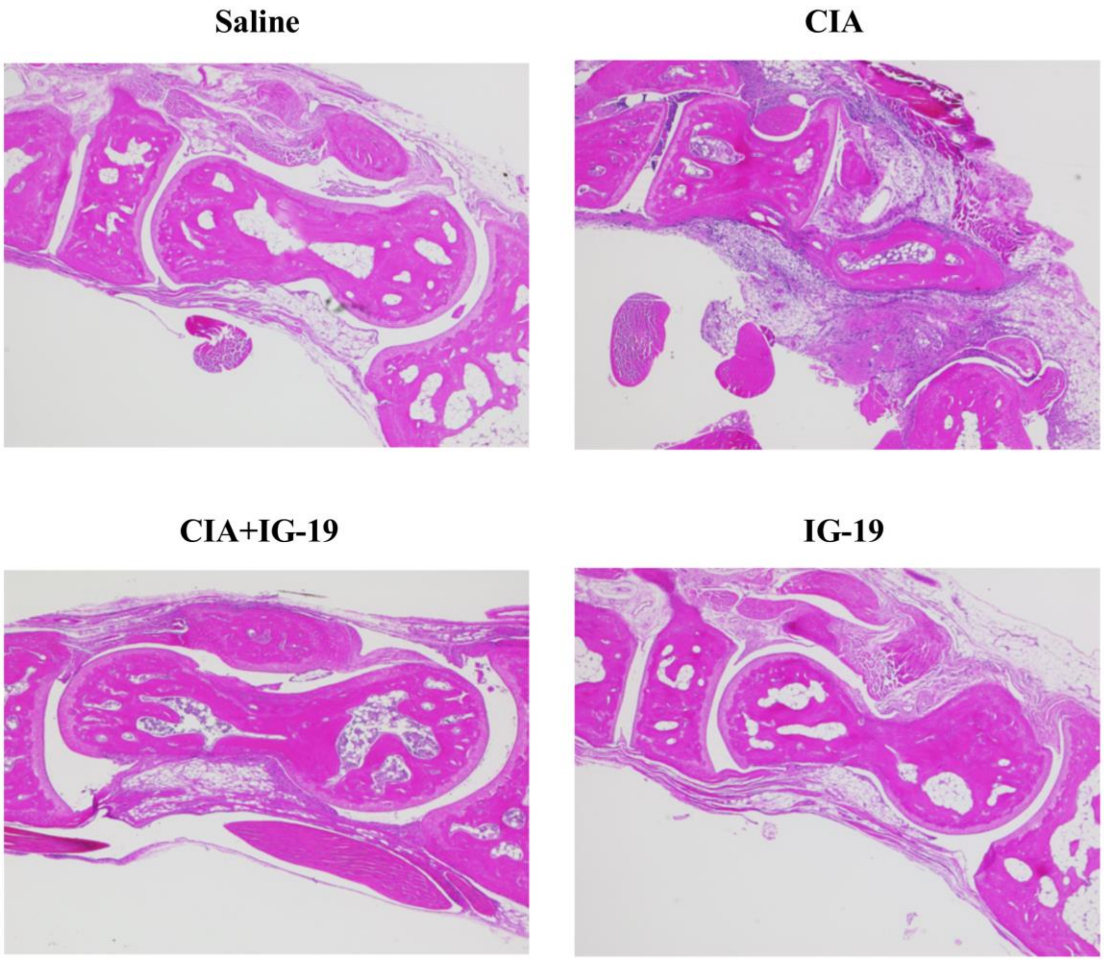

Figure S2: Administration of IG-19 improves the CIA-induced morphological changes of the joint. DBA/1 mice from the CIA mouse model were euthanized by cardiac puncture under anesthesia on day 29 after the initial CII challenge. The joints were deskinned and collected in 10% buffered formalin, decalcified in 10% EDTA and processed for histology. The paraffin embedded sagittal sections (5 µm) of hind ankle joints were stained with H&E to detect the cellular infiltration and joint integrity. Sections from each joint were imaged and scored to obtain cumulative histology scores. Images shown are representative of sections from each group (n = 5 in each group). The images were processed using a Zeiss imager M2 using the Zen 2011 software.

Supplementary Figure 3:

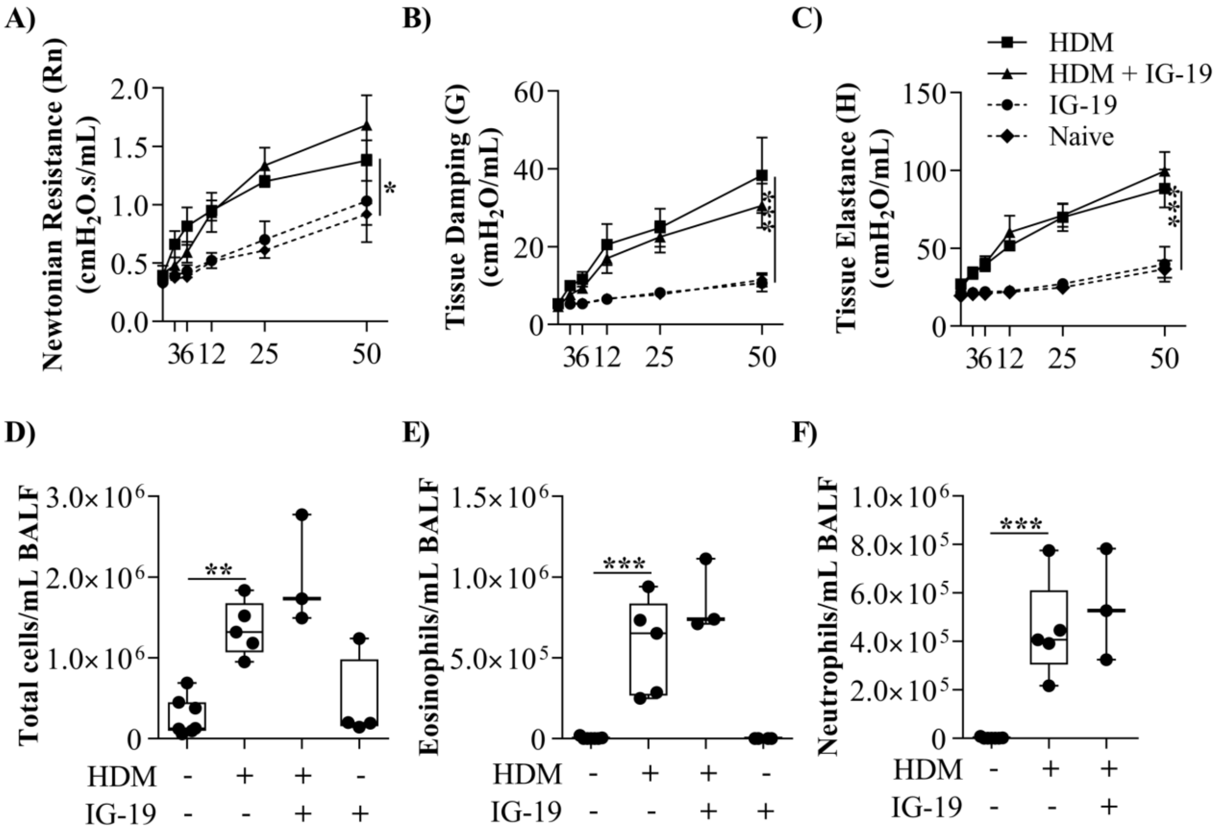

Figure S3: Administration of IG-19 does not improve airway hyper-responsiveness (AHR), and does not suppress immune cell infiltrations in HDM-challenged mice. Female BALB/c (8–10 weeks) mice (n=5 per group) were challenged with 35 µL of whole HDM extract (0.7mg/mL) in saline intranasally for 2 weeks. IG-19 was administered subcutaneously (6mg/kg) three times a week. Lung mechanics was monitored using a flexiVent small animal ventilator, 24 hr after the last HDM challenge. Mice were exposed to nebulized saline (baseline measures) followed by increasing concentrations of nebulized methacholine (3-50mg/mL) and changes in **(A)** Newtonian resistance (Rn), **(B)** tissue damping (G), **(C)** tissue elastance (H) were monitored. Broncheoalveolar lavage fluid (BALF) was collected from all mice 24 hr after the last HDM challenge. **(D)** Total cell, **(E)** eosinophil, and **(F)** neutrophil numbers were assessed. Statistical significance for A, B and C were determined using two-way repeated measures analysis of variance with Tukey's multiple comparisons test. Statistical significance for panels D, E and F was determined using one-way analysis of variance with Tukey's multiple comparisons test (*$p \leq 0.05$, **$p \leq 0.01$, ***$p \leq 0.001$). Asterisks in the figure represent comparison between naïve and the HDM group.

Supplementary Figure 4:

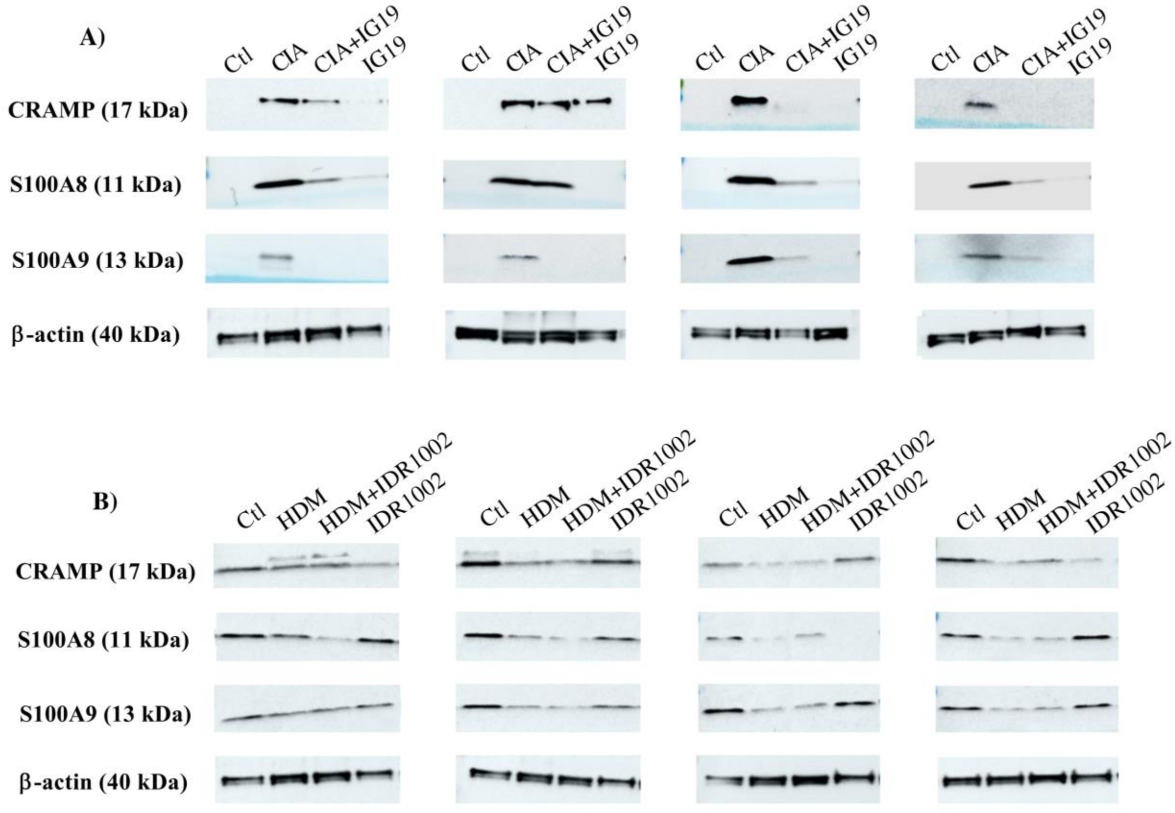

Figure S4: Western blots for abundance of CRAMP, S100A8 and S100A9, with and without IG-19 and IDR-1002 administration. Tissues were obtained from **(A)** joints of the CIA mice, along with CIA mice administered with IG-19 (n=4 per group), and **(B)** lungs of HDM-challenged mice along with HDM-mice administered with IDR-1002 (n=4 per group) were collected. In each model, saline-treated mice were controls (Ctl) and peptide-alone-treated mice were used (n=4 per group). Flash frozen tissues were homogenized and protein concentration was determined using microBCA. Each sample (20 μg protein each) were resolved on NuPage 4-12% Bis-Tris protein gels and transferred onto nitrocellulose membranes. The membranes were probed with specific antibodies for mouse CRAMP, S100A8 and S100A9, and antibody to β-actin (for loading control). The figure includes all 4 independent samples from each group.

Supplementary Figure 5:

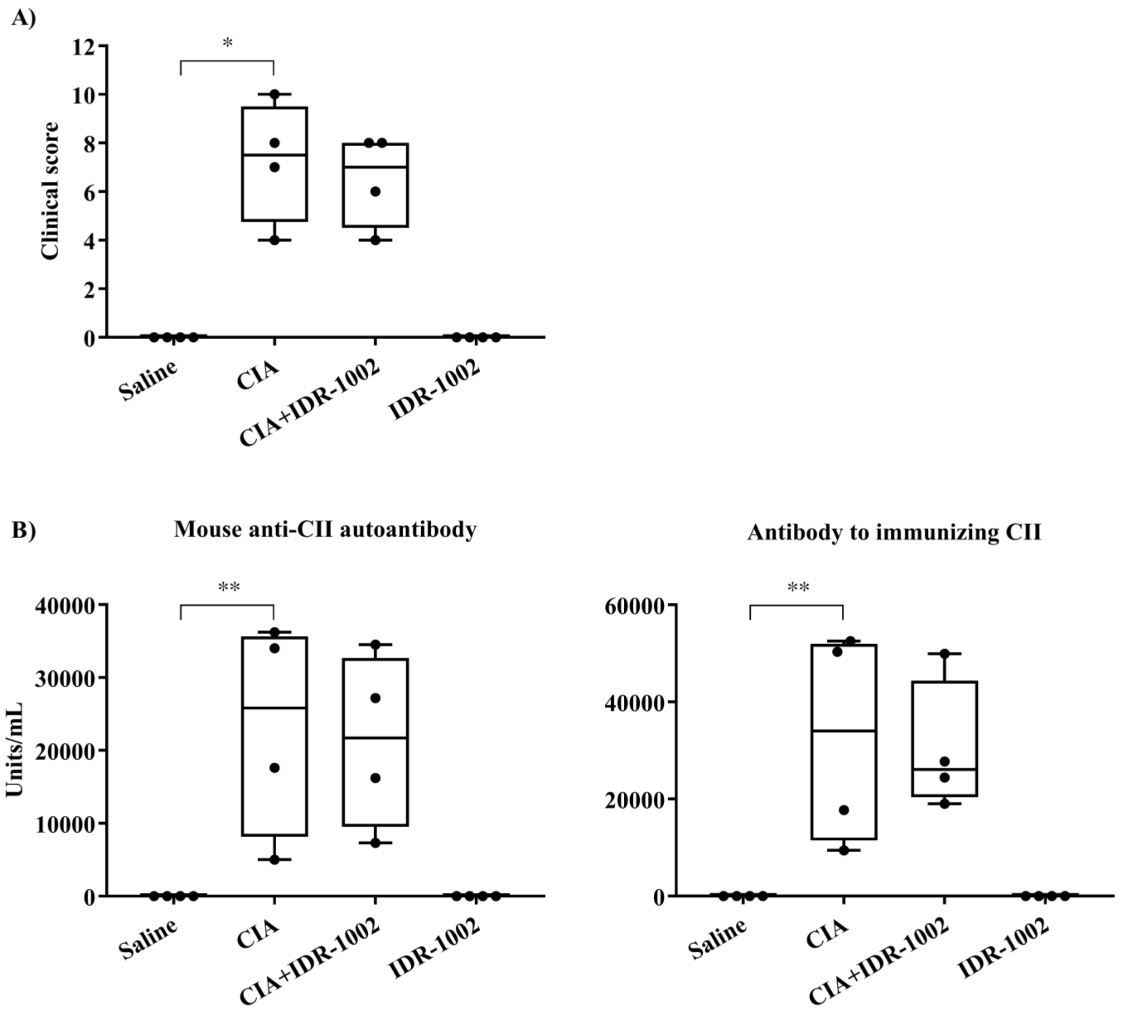

Figure S5: Administration of IDR-1002 does not improve clinical scores and does not suppress anti-collagen antibody levels in CIA mice. DBA/1 male mice (8 weeks) were challenged with chicken CII as the immunizing antigen (n=4), followed by another booster dose of CII on day 21 after the initial CII challenge. The peptide IDR-1002 was administered (s.c) starting from day 20 (one day before boost) and subsequently every 48 h until the end of the study**.** Mice were monitored for disease severity and scored to assign clinical scores every alternate day from day 30 to day 54. The scoring method is described in the methods section. Mice were euthanized by cardiac puncture under anesthesia on day 55 after the initial CII challenge, and blood was collected for serum. **(A)** Clinical scores on day 54 (at the end of the experiment). **(B)** Serum concentrations of anti-mouse collagen type II autoantibodies and anti-chicken collagen type II antibodies (to immunizing antigen) were determined by ELISA (Chondrex Inc. USA). GraphPad Prism 7.05 software was used for statistical analyses. Kruskal–Wallis One-way ANOVA followed by Dunn's multiple comparison test was used to determine the significance (*$p \leq 0.05$, **$p \leq 0.01$, ***$p \leq 0.001$).
